# Adverse events of immune checkpoint therapy alone versus when combined with vascular endothelial growth factor inhibitors: a pooled meta-analysis of 1735 patients

**DOI:** 10.3389/fonc.2023.1238517

**Published:** 2024-01-04

**Authors:** Iuliia Kovalenko, Wern Lynn Ng, Yimin Geng, Yinghong Wang, Pavlos Msaouel, Shailender Bhatia, Petros Grivas, Raed Benkhadra, Omar Alhalabi

**Affiliations:** ^1^ Internal Medicine Department, UPMC Harrisburg, Harrisburg, PA, United States; ^2^ Department of Genitourinary Medical Oncology, University of Texas MD Anderson Cancer Center, Houston, TX, United States; ^3^ Fred Hutchinson Cancer Center, Department of Hematology and Oncology, University of Washington, Seattle, WA, United States; ^4^ Department of Hematology and Oncology, University of Texas Health Science Center at San Antonio, San Antonio, TX, United States

**Keywords:** cancer, immunotherapy, toxicity, adverse events, immune checkpoint inhibitor, vascular endothelial - growth factor

## Abstract

**Background:**

Combining immune checkpoint therapy (ICT) and vascular endothelial growth factor inhibitors (VEGFi) may result in increased treatment-related and immune-related adverse events (TRAEs and irAEs) compared to ICT alone. This metanalysis was conducted to identify prospective phase II or III clinical studies that evaluated the toxicity profile of ICT + VEGFi compared to ICT alone.

**Methods:**

A systematic search was performed across all cancer types and major databases until August 10, 2022, and screening was done by two independent investigators. Inclusion criteria included phase 2 or 3 studies with at least one arm of patients treated with combination therapy and one arm treated with monotherapy. Adverse event data were pooled using a restricted maximum likelihood fixed effects model, and heterogeneity using Cochran’s Q (chi-square) test.

**Results:**

7 out of 9366 studies met the inclusion criteria, and 808 and 927 patients were treated with ICT monotherapy and a combination of ICT with VEGFi, respectively. Only one study reported irAEs, so the analysis was restricted to TRAEs. The total number of TRAEs was significantly higher in the ICT + VEGFi group (RR:1.49; 95% CI 1.37 -1.62; p=1.5×10-21), and more frequent treatment withdrawals were attributed to TRAEs (RR:3.10; 95% CI 1.12-8.59; p=0.029). The highest TRAE effect size increases noted for rash (RR 6.50; 95% CI 3.76 – 11.25; p=2.1×10-11), hypertension (RR:6.07; 95% CI 3.69–10.00; p=1.3×10-12), hypothyroidism (RR:5.02; 95% CI 3.08 – 8.19; p=8.9×10-11), and diarrhea (RR:4.94; 95% CI 3.21–7.62; p=3.8×10-13). Other significantly more frequent TRAEs included nausea, anemia, anorexia, and proteinuria.

**Conclusion:**

Combination therapy with ICT and VEGFi carries a higher risk of certain TRAEs, such as rash, hypertension, hypothyroidism, diarrhea, nausea, anorexia, and proteinuria, compared to ICT monotherapy. More granular details on the cause of AEs, particularly irAEs, should be provided in future trials of such regimens.

## Background

Immune checkpoint inhibitors therapy (ICT) combined with vascular endothelial growth factor inhibitors (VEGFi) are now established standard of care regimens across diverse malignancies such as renal cell carcinoma, endometrial cancer and hepatocellular carcinoma (1–4). However, there is limited knowledge about the potential synergistic toxicities between combination of ICT with VEGFi when compared to ICT alone (5–9). Prior studies have aimed to describe the general safety profile of this combination. For example, Tao et al. conducted a meta-analysis to evaluate the efficacy and toxicity of ICT and VEGFi in comparison to VEGFi alone in patients with renal cell carcinoma (RCC). The study included six randomized clinical trials (RCTs) and analyzed dose-limiting adverse events (AEs) related to RCC treatment. The results showed an increased risk of any-grade treatment-related adverse events (TRAEs), such as hypertension, arthralgia, and proteinuria, in the combination group compared to the VEGFi alone group. However, the risk of some TRAEs, such as hand-foot skin reaction (HFSR) [RR = 0.47, 95% CI: 0.28– 0.79], stomatitis (RR = 0.71, 95% CI: 0.56–0.91), and dysgeusia (RR = 0.42, 95% CI: 0.26–0.68), was lower in the combination group (1). A similar study by He et al. included six RCTs and evaluated the safety and efficacy of ICT and VEGFi combination therapy compared to VEGFi alone in the treatment of RCC. The results showed no significant difference in grade 3 or higher TRAEs between the two groups (2). A small study by Rizzo et al. aimed to evaluate the risk of gastrointestinal (GI) toxicities of a combination of immunotherapy with tyrosine kinase inhibitors (TKIs) compared to sunitinib alone. The meta-analysis of four RCTs showed an increased risk of selected GI TRAEs, such as diarrhea and decreased appetite, in the combination group, while the risk of nausea was higher in the sunitinib group (3). Several early-phase trials and retrospective studies also aimed to look at the efficacy and toxicity of ICT and TKIs, such as epidermal growth factor receptor inhibitors (EGFRi), as a combination therapy as well as sequential therapy. These studies results exhibited discrepancy with some of them suggesting increased toxicity of combination and sequential therapies while others reported acceptable safety profiles (10–13). Discrepant results from these studies create a knowledge gap regarding the risk of added toxicities of ICT/VEGFi combination regimens. Moreover, the results of meta-analyses by Abdelhafeez et al. and Da et al. showed an expected increase overall risk of irAEs with the use of two combined immune checkpoint inhibitors as compared to one (14, 15). However, little is known regarding the risk of immune- related AEs (irAEs) of ICT/VEGFi combination therapy. Our hypothesis was that there are added immune and other toxicities when using combination of ICT/VEGFi as compared to ICT alone. To answer this question, we conducted a meta-analysis of reported or published studies with toxicity data of ICT/VEGFi combination therapy as compared to ICT alone.

## Methods

### Data sources and search strategies

We performed a systematic search in Ovid MEDLINE, Ovid Embase, Clarivate Web of Science and Wiley Cochrane Library from the inception of the databases to August 10, 2022. Search structures, subject headings, and keywords were tailored to each database by a medical research librarian (YG). The following concepts were searched using subject headings and keywords as needed, “cancer”, “neoplasm”, “immunotherapy”, “checkpoint inhibitor”, “cytotoxic t- lymphocyte-associated antigen 4”, “programmed cell death 1”, “programmed cell death ligand 1”, “vascular endothelial growth factor”, “VEGF inhibitor”, “vascular endothelial growth factor receptor”, “anti-vascular”, “anti-VEGF”, “anti-angiogenic”, “angiogenesis inhibitor”, “ipilimumab”, “tremelimumab”, “pembrolizumab”, “nivolumab”, “spartalizumab”, “cetrelimab”, “atezolizumab”, “durvalumab”, “avelumab”, “cemiplimab”, “monalizumab”, “aflibercept”, “bevacizumab”, “ranibizumab”, “brolucizumab”, “conbercept”, “pazopanib”, “sunitinib”, “sorafenib”, “regorafenib”, “cabozatinib”, “lenvatinib”,”ponatinib”, “axitinib”, “tivozanib”, “ramucirumab”, “vandetanib”, and “sitravatinib”. The search terms were combined by “or” if they represented the similar concept, and by “and” if they represented different concepts. Database search strategies are detailed in the **Supplementary Tables S1**–**S4**.

### Eligibility criteria

In determining eligibility for our review, we established several inclusion criteria. The studies had to be phase 2 or 3, reported in English, and include at least one arm of adult patients treated with a combination of ICT and VEGFi, as well as one arm treated with ICT monotherapy. Additionally, the studies had to report outcomes related to TRAEs and/or IRAEs. We excluded non-comparative and non-original studies, and studies that did not report AEs. Retrospective studies were also excluded from our analysis because we aimed to ensure the integrity and reliability of our data in relation to CTCAE criteria. By using prospectively collected data with CTCAE criteria, we aimed to minimize the potential for recall bias, which can occur when relying on retrospective data. Abstracts without a full text that met our inclusion criteria were still included in the analysis.

### Study selection

The study selection process was carried out by two independent reviewers (IK and LW) who screened all titles and abstracts based on the defined inclusion and exclusion criteria. The full text of relevant references were obtained and evaluated by the same two reviewers. In case of any discrepancy in selection, a third reviewer (OA) was involved to resolve it.

### Data extraction

Data extraction was performed by two independent reviewers (IK and LW) using Microsoft Excel. Any discrepancies in data extraction were resolved by two other independent reviewers (OA and RB). The following variables were collected from each study: study characteristics, participant characteristics, intervention details, and the outcomes of interest, which included the total sample size and the number of events in each group.

### Outcomes of interest

The outcomes of interest included treatment-related adverse events (TRAEs) and immune-related adverse events (irAEs). The categorization of TRAEs and irAEs was predicated upon definitions provided by the individual studies included. When a study explicitly defined an event as an irAE, we categorized the data accordingly. In the absence of such specific categorization, events were defaulted to treatment related. This methodology ensured consistency and minimized interpretative biases in our analysis. TRAEs included symptoms such as diarrhea, rash, HFSR, fever, dry mouth, pruritus, conjunctivitis, hypomagnesemia, dysphonia, nausea, increased creatinine, increased ALT, increased AST, increased bilirubin, increased lipase, fatigue, asthenia, hypertension, anorexia, weight loss, mucositis, decreased platelet count, thyroid dysfunction, thyroiditis, hypothyroidism, anemia, increased TSH, decreased lymphocytes, decreased neutrophils, headache, infection, proteinuria, arthralgia, seizures, hyperglycemia, infusion reaction, and more. Similarly, irAEs included symptoms such as abdominal pain, increased ALP, increased ALT, increased AST, increased bilirubin, cerebral edema, colitis, conjunctivitis, increased creatinine, arthralgia, diarrhea, dyspnea, hypothyroidism, hyperthyroidism, infusion reaction, hyperglycemia, myalgia, rash, and others. The Common Terminology Criteria for Adverse Events v3.0 was used to grade the adverse events. The events were considered for analysis if they were reported similarly by at least two studies.

### Quality assessment

The methodologic quality was assessed using the Cochrane risk-of-bias tool for randomized trials. The risk of bias was only assessed for published full-length articles. Two reviewers independently (IK and LW) assessed trial quality of studies by examining several components: randomization process, deviations from intended interventions, missing outcome data, selective reporting, funding and any other potential source of bias. Any conflicts were resolved by consensus. The quality of the studies is represented in the **Table 1**.

**Table 1:** Traffic light plot showing the risk of bias of the two completed studies.

Author, Year	Risk of bias domains						
	D1	D2	D3	D4	D5	D6	D7
Nayak, 2020 (16)	Low	Low	Low	Low	Some Concerns	Low	Some Concerns
Lheureux, 2020 (17)	Low	Low	Low	Low	Some Concerns	Low	Low

Domains:

D1: Overall ROB.

D2: ROB from randomization process.

D3: ROB due to deviations from intended interventions.

D4: ROB due to missing outcome data.

D5: ROB in measurement of outcomes.

D6: ROB in selection of the reported results.

D7: Other (funding, conflict of interest).

### Data analysis

For the adverse events, we calculated relative risk along with 95% confidence intervals and we pooled the effect estimates across the studies following the restricted maximum likelihood fixed heterogeneity. For the assessment of heterogeneity, we used Cochran’s Q (chi square) test, P value <0.1 is considered statistically significant and I 2 ≥ 50% suggested substantial heterogeneity. Forest plots were constructed to illustrate the results of the meta- analysis. Statistical analyses were completed using R version 4.2.2 (R Core Team, 2020).

### Ethics statement

This study was exempt from Institutional Review Board review as it involved the analysis of existing publicly available data.

### PRISMA statement

This study was conducted in accordance with the PRISMA guidelines (Preferred Reporting Items for Systematic Reviews and Meta-Analyses). The PRISMA checklist was used to guide the study and is available upon request (**Table S5** in the appendix) (18, 19).

## Results

### Study characteristics

**Figure 1:**
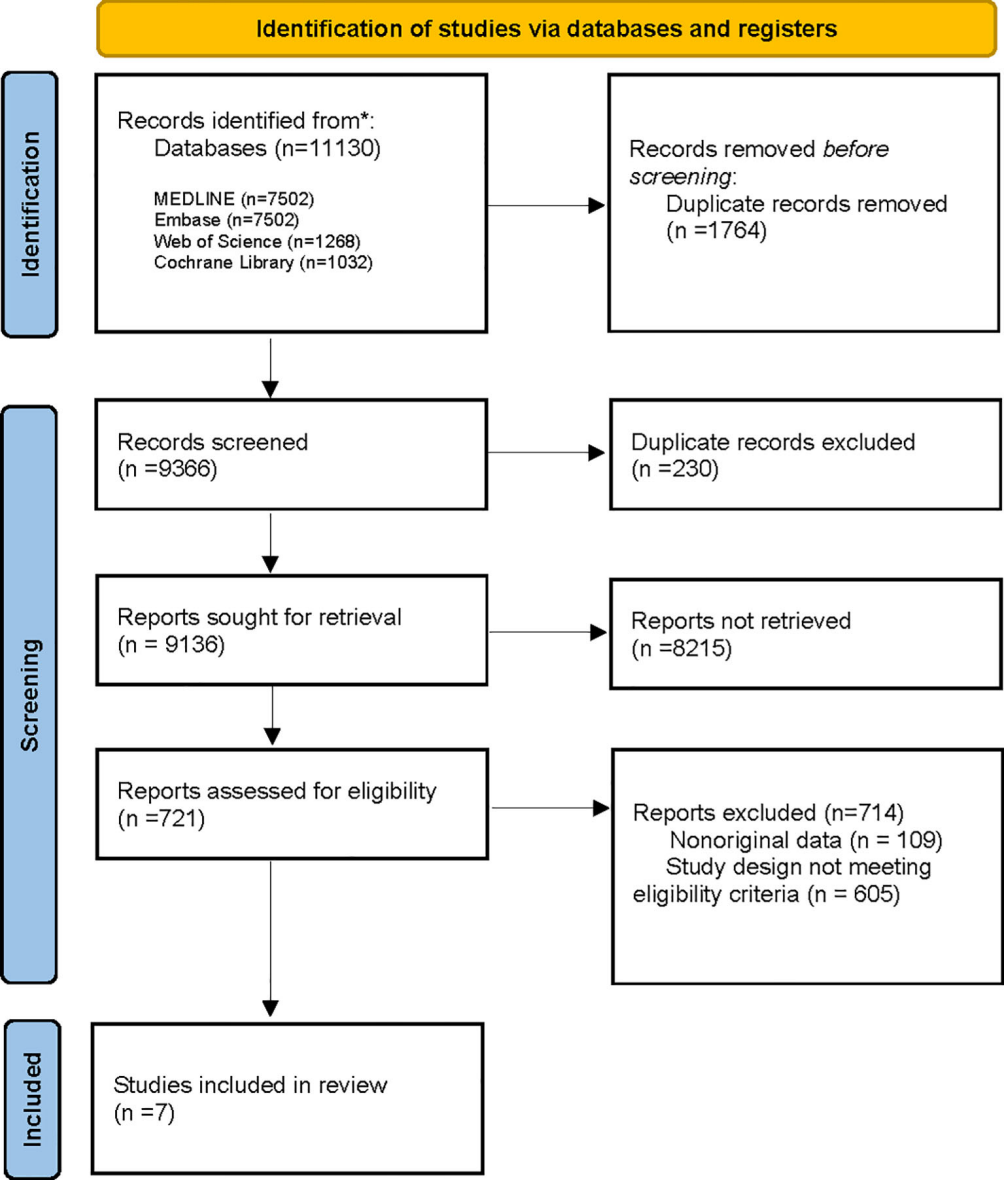
Flowchart demonstrating the process of study selection (7).

A total of 11,130 potential titles and abstracts were identified through the electronic search strategy, with 32 duplicates removed internally and 1764 duplicates removed through the assistance of a medical research librarian (YG). The remaining 9366 studies underwent primary screening, and 721 full-text articles or abstracts were evaluated for eligibility (as shown in **Figure 1**) (19)]. After secondary screening, seven studies were included in the analysis, involving a total of 808 patients treated with ICT monotherapy and 927 patients treated with a combination of ICT and VEGFi. The characteristics of the included studies are summarized in **Table 2**. Further details on the baseline characteristics of the studies can be found in **Table S6** in the appendix.

**Table 2:** Baseline characteristics of patients among the included studies.

Year	Author	Trial name	NCT number	Phase	Conference	Full manuscript (FM) vs Abstract (A)	Number of patients	Median age	Males, %	Cancer type	ICT arm	ICT + VEGFi arm
2018	Bendell et al. (20)	IMblaze370	NCT02788279	3		FM	363	58		colorectal	atezolizumab	Atezolizumab + cobimetinib
2017	McDermott et al. (21)	IMmotion150	NCT01984242	2	2017 Genitourinary Cancers Symposium	A	305			RCC	atezolizumab	Atezolizumab + bevacizumab
2020	Lonardi et al. (22)	CARACAS	NCT03944252	2	2020 ASCO Annual Meeting	A	60	63	31.6	Squamous cell anal carcinoma	avelumab	Avelumab + cetuximab
2022	Loriot et al. (23)	LEAP-011	NCT03898180	3	2022 ASCO Genitourinary Cancers Symposium	A	441			Urothelial carcinoma	pembrolizumab	Pembrolizumab + lenvatinib
2020	Nayak et al. (16)		NCT02337491	2		FM	80	53	67.5	Glioblastoma	pembrolizumab	Pembrolizumab + bevacizumab
2021	Yang et al. (24)	LEAP-007	NCT03829332	3	The ESMO Immuno-Oncology Congress 2021	A	623	66		Non-small cell lung cancer	pembrolizumab	Pembrolizumab + lenvatinib
2020	Lheureux et al. (17)		NCT03367741	2	2020 ASCO Annual Meeting	A	82			Endometrial carcinoma	nivolumab	Nivolumab + carbozatinib

ICT, immune checkpoint inhibitors therapy; VEGFi, vascular endothelial growth factor inhibitors.

### Adverse events

**Figure 2:**
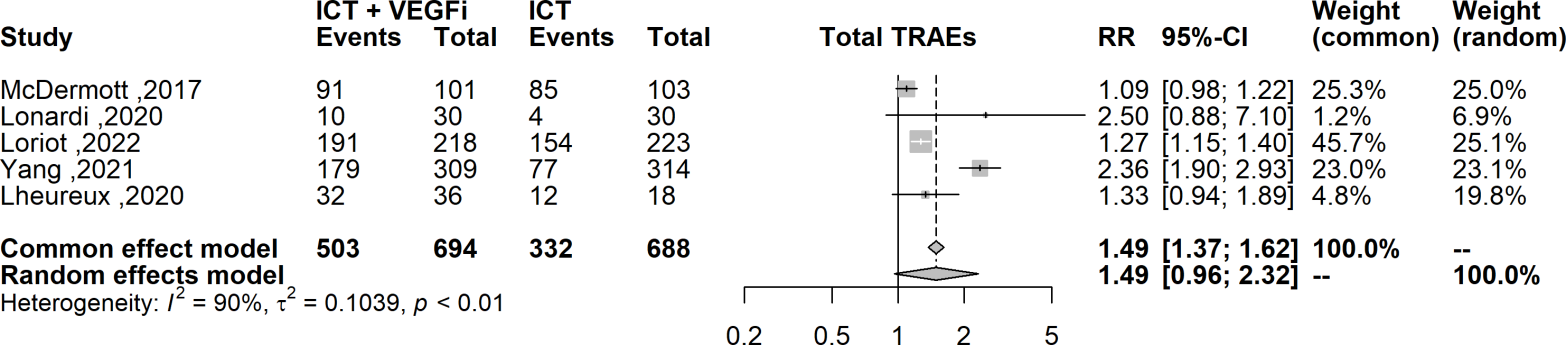
Forest plot comparing the risk of any grade TRAEs between ICT vs combination of ICT with VEGFi.

**Figure 3:**
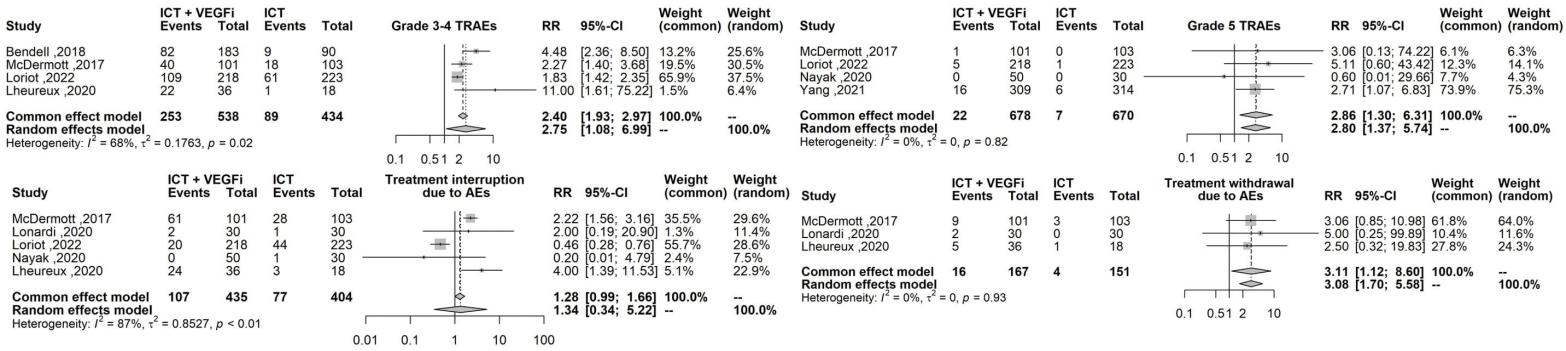
Forest plot comparing the risk grade 3-4 TRAEs, grade 5 TRAEs, the risk of treatment interruption and treatment withdrawal between ICT vs combination ICT with VEGFi.

**Figure 4:**
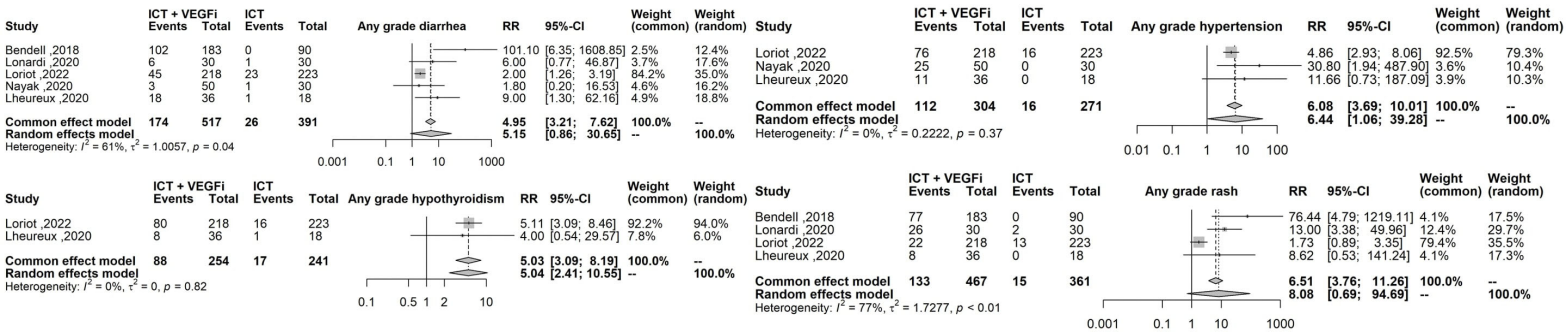
Forest plot comparing the risk of the highest effect size TRAEs: sny grade diarrhea any grade hypertension, any grade hyphothyroidism, any grade rash.

**Figure 5:**
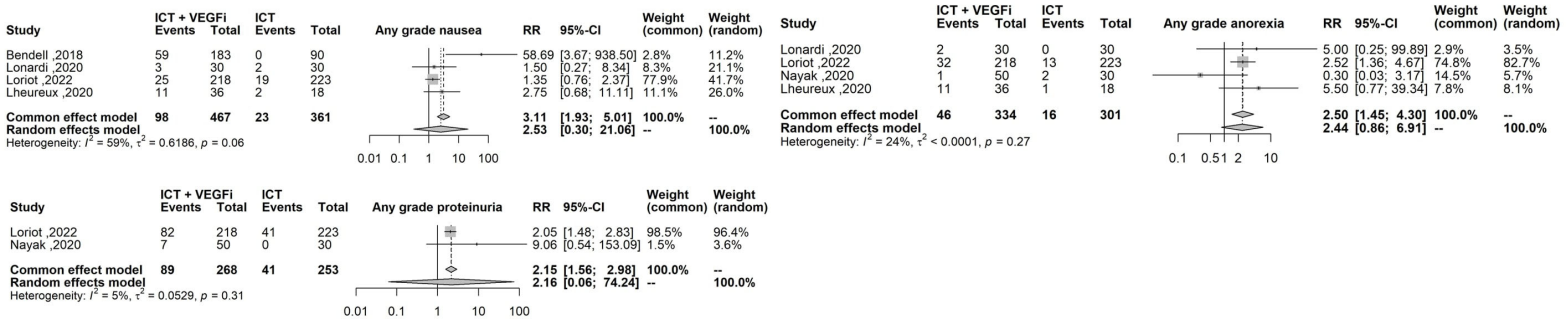
Forest plot comparing risk of any grade nausea, any grade proteinuria, any grade anorexia.

**Figure 6:**
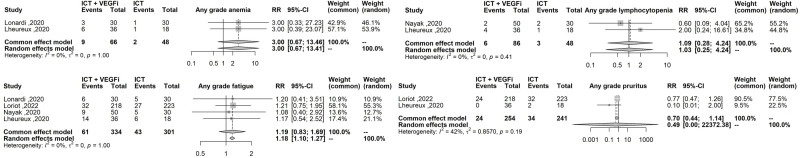
Forest plot comparing the risk of any grade anemia, any grade lymphopenia, any grade fatigue, any grade pruritus.

Only one of the studies reported immune-related adverse events (irAEs). As a result, the analysis was restricted to treatment-related adverse events (TRAEs). The TRAEs included in the analysis were anemia, anorexia, diarrhea, fatigue, hypertension, hypothyroidism, lymphopenia, nausea, proteinuria, pruritus, and rash of any grade. The total number of TRAEs was significantly higher in the group receiving the combination of ICT and VEGFi (relative risk [RR] 1.49; 95% CI 1.37 - 1.62; p=1.5×10-21). The rate of grade 5 TRAEs was higher in the combination group (RR 2.86; 95% CI 1.29 - 6.31; p=0.0091). Treatment withdrawals due to TRAEs were also higher in the combination group (RR 3.10; 95% CI 1.12 - 8.59; p=0.029). However, the signal for an increased rate of treatment interruptions due to TRAEs was weaker (RR 1.28, 95% CI 0.99 - 1.65; p=0.057). The increased risk of TRAEs was significantly higher in the combination group for the following events: anorexia (RR 2.49; 95% CI 1.45 – 4.30; p=9.5×10-4), diarrhea (RR 4.94; 95% CI 3.21 – 7.62; p=3.8×10-13), hypertension (RR 6.07; 95% CI 3.69 – 10.00; p=1.3×10-12), hypothyroidism (RR 5.02; 95% CI 3.08 – 8.19; p=8.9×10-11), nausea (RR 3.10; 95% CI 1.93 – 5.00; p=3.1×10-6), proteinuria (RR 2.15; 95% CI; p=3.6×10-6), and rash (RR 6.50; 95% CI 3.76 – 11.25; p=2.1×10- 11). However, the risk was inconclusive for certain TRAEs such as anemia (RR 3.00, 95% CI 0.66 - 13.45; p=0.15), lymphopenia (RR 1.08, 95% CI 0.27 - 4.23; p=0.9), fatigue (RR 1.18, 95% CI 0.82 - 1.69; p=0.35), and pruritus (RR 0.70, 95% CI 0.43 - 1.13; p=0.15). The list of TRAEs and their corresponding effect sizes can be found in **Table 3**. Forest plots demonstrating our results are presented in **Figures 2**–**6**.

**Table 3:** Summary of the safety findings of the combination of ICT with VEGFi versus ICT alone.

Outcome	Number of studies	Total combined	Total events combined	Total individual	Events individual	RR	95% CI	P value
Total % of TRAEs	5	694	503	688	332	1.49	1.37 -1.62	1.5×10^-21^
Grade 3-4 TRAEs	4	538	253	434	89	2.40	1.93 - 2.97	1.7×10^-15^
Grade 5 TRAEs	4	678	22	670	7	2.86	1.29 - 6.31	9.1×10^-3^
Treatment interruption due to TRAEs	5	435	107	404	77	1.28	0.99 - 1.65	5.7×10^-2^
Treatment withdrawal due to TRAEs	3	167	16	151	4	3.10	1.12 - 8.59	2.9×10^-2^
Any grade anemia	2	66	9	48	2	3.00	0.66 - 13.45	1.5×10^-1^
Any grade anorexia	4	334	46	301	16	2.49	1.45 - 4.30	9.5×10^-4^
Any grade lymphopenia	2	86	6	48	3	1.08	0.27 - 4.23	9×10^-1^
Any grade diarrhea	5	517	174	391	26	4.94	3.21 - 7.62	3.8×10^-13^
Any grade fatigue	4	334	61	301	43	1.18	0.82 - 1.69	3.5×10^-1^
Any grade hypertension	3	304	112	271	16	6.07	3.69 - 10.00	1.3×10^-12^
Any grade hypothyroidism	2	254	88	241	17	5.02	3.08 - 8.19	8.9×10^-11^
Any grade nausea	4	467	98	361	23	3.10	1.93 - 5.00	3.1×10^-6^
Any grade proteinuria	2	268	89	253	41	2.15	1.55- 2.97	3.6×10^-6^
Any grade pruritus	2	254	24	241	34	0.70	0.43 - 1.13	1.5×10^-1^
Any grade rash	4	467	133	361	15	6.50	3.76 - 11.25	2.1×10^-11^

RR, relative risk; CI, confidence interval; ICT, immune checkpoint inhibitors; VEGFi, vascular endothelial growth factor inhibitor.

## Discussion

This systematic review and meta-analysis evaluated the safety of combining ICT with VEGFi compared with ICT alone in adult patients with cancer, incorporating data from 7 studies with a total population of 1735 patients. To the best of our knowledge, this is the first systematic review and meta-analysis that compared toxicity of a combination of ICT with VEGFi as compared to ICT monotherapy across various cancer types. Our study showed that the combination therapy was associated with a significantly increased risk of treatment-related toxicity, risk of death, and treatment discontinuation due to adverse events (AEs). The results demonstrated that the combination therapy increased the risk of anorexia, diarrhea, hypertension, hypothyroidism, nausea, proteinuria, and rash. These AEs are also commonly encountered in monotherapies with VEGFi (25, 26).

The impact of combining ICT with VEGFis on the incidence of adverse events has been a subject of heightened interest. Our findings show a notable association between the two, which is in alignment with the COSMIC-312 trial results. In this pivotal trial that explored the outcomes of patients with renal cell carcinoma (RCC) treated with cabozantinib combined with nivolumab and ipilimumab versus nivolumab and ipilimumab alone, a stark difference in the occurrence of high-grade adverse events was observed. Specifically, the group treated with the combination therapy experienced grade 3-4 adverse events with greater frequency (79% *vs* 56%). The nearly two-fold increase in ICI-related toxicity in the combination therapy group compared to the monotherapy group in the COSMIC-312 trial is a poignant revelation. Such findings underscore the necessity of understanding the potential synergistic effects on toxicity when combining ICIs with other targeted agents. While combination therapies often seek to exploit complementary mechanisms of action to achieve superior antitumor efficacy, they may also inadvertently amplify the risk of severe adverse events. This amplification in toxicity could result from the simultaneous modulation of multiple pathways, leading to unforeseen interactions that heighten patient risk (27).

To date, three meta-analyses have been conducted to evaluate the safety profile of a combination therapy of ICT and VEGFi. All three studies compared the combination therapy to VEGFi monotherapy and are summarized in **Table 4**. The study by He et al. noted an increased risk of Grade 3-4 TRAEs, which is consistent with the results of our study. However, there was no analysis performed on the breakdown of AEs (2). The meta-analysis by Tao et al. only evaluated specific AEs that are monitored in RCC, limiting the scope of their results. Nonetheless, they found an increased risk of hypertension, proteinuria, and rash with ICT plus VEGFi combination compared with VEGFi alone (1), which we also noted in our comparison between ICT plus VEGFi versus ICT monotherapy. The meta-analysis by Rizzo et al. aimed to compare the combination of ICT with TKIs to TKI monotherapy in terms of the risk of gastrointestinal toxicity. They noted an increased risk of diarrhea and decreased appetite (referred to as anorexia in our study) in the ICT and TKI combination group compared with TKI monotherapy (3). Because ICT monotherapy is now a standard option across different malignancies, our study provides additional context that can inform clinical decision-making and current practice patterns by comparing the TRAEs with ICT plus VEGFi versus ICT monotherapy. Increased awareness of the specific TRAE risks associated with the combination of ICT with VEGFi will help to monitor, prevent, and treat treatment toxicities in a timely manner (14, 15). However, further data is needed to fully understand the risk of irAEs with ICT plus VEGFi versus ICT monotherapy.

**Table 4:** Summary of meta-analyses results evaluating the risk of toxicities of ICT and VEGFi combination therapy.

Author	Number of studies included	Treatment	Cancer type	Results (combination therapy vs monotherapy)
He et al.	6	ICT + VEGFi vs VEGFi	RCC	Equal risk of Grade 3-4 TRAEs.
Tao et al.	6	ICT + VEGFi vs VEGFi	RCC	Increased risk of all-grade hypertension, arthralgia, rash, proteinuria, grade 3–5 arthralgia, and proteinuria. Equal risk of grade 3-4 hypertension, grade 3-5 rash. Decreased risk of HFSR, stomatitis, dysgeusia.
Rizzo et al.	4	ICT + TKIs vs TKI	RCC	Increased risk of all-grade diarrhea, grade 3–4 decreased appetite. Decreased risk of all-grade nausea

ICT, immune checkpoint inhibitor therapy; VEGFi, vascular endothelial growth factor inhibitor; TKI, tyrosine kinase inhibitor; HFSR, hand-foot skin reaction.

The amalgamation of ICT with VEGFi introduces a complex interplay of enhanced therapeutic potential against the backdrop of augmented toxicities, a challenge particularly evident in kidney cancer. Our meta-analysis, delineating the adverse event profile of ICIs in isolation versus their concomitant administration with VEGFis, underscores this potential enhanced toxicity. Notably, in the realm of kidney cancer, most of trials that combine immunotherapy often employ doses lower than when used in monotherapy. This dose reduction, in part, stems from concerns over enhancing toxicity. Such strategies highlight the importance of an intricate balancing act to maintain clinical sustainability. A potential avenue to sustain treatment efficacy while minimizing adverse effects is to employ reduced drug doses, coupled with individualized therapeutic modulation, informed by early surveillance and predictive biomarkers. By harnessing insights from our meta-analysis, clinicians can judiciously navigate the nexus of potency and safety, optimizing the therapeutic window of these combinatorial regimens.

Our study has several limitations. Only 2 peer-reviewed publications were included, and most studies were available only as abstracts, making it difficult to assess the risk of bias. Additionally, our study assumed that the type of cancer does not impact immunotherapy toxicity, while some studies have suggested that the risk of immunotherapy toxicity may be higher in certain types of cancer, such as lung cancer (28, 29). Our analysis only included one RCT that evaluated the safety profile of ICT versus ICT with VEGFi combination therapy in non-small cell lung cancer (24). Furthermore, we had a limited number of studies, with only one study reporting irAEs, an outcome of interest which we were not able to include in our meta-analysis. The publication of the full manuscript reports of trials is warranted, with particular focus on the risk of irAEs with ICT and VEGFi combination therapy compared with ICT monotherapy. One notable limitation of our study pertains to the absence of a sensitivity analysis that would account for both the diversity of pathologies and the consideration of prior treatments. Our dataset was constrained in its ability to permit such an analysis due to the paucity of subgroup data. Only three studies within our collection provided details on previous treatment lines, a factor known to potentially influence TRAE profiles. Additionally, while it is well-documented in prior research that patients with melanoma and non-small cell lung cancer (NSCLC) exhibit a heightened risk of TRAEs upon sensitivity analysis, our meta-analysis did not encompass patients with melanoma and incorporated data from just one study addressing NSCLC (15). Such omissions and data limitations could curtail the broader applicability and comprehensiveness of our findings in the oncological realm.

## Conclusion

We found that ICT plus VEGFi combinations yield an increased risk of specific treatment-related adverse events (TRAEs) compared to ICT alone. Healthcare providers should be aware of the elevated risks for specific TRAEs when using the ICT + VEGFi combination therapy, including rash, hypertension, hypothyroidism, diarrhea, nausea, anorexia, and proteinuria. Further studies are necessary to fully understand the risk of irAEs associated with this combination therapy and provide more granular details on the causes of AEs.

## Author contributions

IK: conception; performance of work; interpretation of data; writing the article. WL: performance of work; interpretation of data; writing the article. YG: conception; performance of work. PM: interpretation of data; writing the article. RB: conception; performance of work; interpretation of data; writing the article. OA: conception; performance of work; interpretation of data; writing the article. PG: interpretation of data; drafting the work; provided final approval of the version to be published. All authors contributed to the article and approved the submitted version.
