# Supplementary material for: Adverse events of immune checkpoint therapy alone versus when combined with vascular endothelial growth factor inhibitors: a pooled meta-analysis of 1735 patients
Source: Front Oncol. 2024 Jan 4;13:1238517. doi: 10.3389/fonc.2023.1238517 (PMC10796151; doi:10.3389/fonc.2023.1238517)
Supplement: Supplementary file 1 [file Table_1.pdf]

Supplementary tables

Table S1. Ovid MEDLINE search strategy

#	Searches
1	exp Neoplasms/
2	(cancer* or carcinom* or tumor* or tumour* or neoplas* or malignan* or metasta* or myeloma* or leuk?emia* or lymphoma* or sarcoma* or melanoma* or " myelodysplastic syndrome*" or "stem cell transplant*").ti,ab,kf.
3	1 or 2 [Cancer]
4	("immunotherapy" or "immune therapy" or "immunologic therapy" or "immune checkpoint therapy").ti,ab,kf.
5	(checkpoint adj3 (inhibitor* or modulator* or antibod* or block*)).ti,ab,kf,rn.
6	(checkpoint and (inhibitor* or modulator* or antibod* or block*)).nm.
7	(immun* adj3 checkpoint adj3 (inhibitor* or modulator* or antibod* or block*)).ab.
8	(("cytotoxic T lymphocyte associated" adj3 "4") or "CTLA 4" or CTLA4).ti,ab,kf,rn.
9	("Cytotoxic t-lymphocyte antigen" adj3 "4").ti,ab,kf,rn.
10	("Cytotoxic t-lymphocyte antigen" or "cytotoxic T lymphocyte associated").nm.
11	(ipilimumab or Yervoy).mp.
12	(tremelimumab or ticilimumab*).mp.
13	("Programmed Cell Death 1" or PD1 or "PD 1").ti,ab,kf,rn,nm.
14	(pembrolizumab or keytruda or lambrolizumab).mp.
15	(nivolumab or opdivo).mp.
16	(spartalizumab* or cetrelimab* or JNJ-63723283).mp.
17	("programmed death ligand 1" or "PD L1" or PDL1 or "PDL-1").ti,ab,kf,rn,nm.
18	(atezolizumab or Tecentriq or durvalumab or imfinzi or avelumab or Bavencio or cemiplimab or libtayo or REGN2810 or "REGN 2810").mp.
19	monalizumab.mp.
20	or/4-19 [immunotherapy checkpoint inhibitors]
21	3 and 20
22	limit 21 to english language
23	(animals not (humans and animals)).sh.
24	22 not 23
25	(mice or mouse or murine or rat or rats or rodent or cells or "in vitro" or "cell line").ti.
26	24 not 25 [Remove animal or in vitro studies]
27	exp Vascular Endothelial Growth Factors/
28	(("vascular endothelial growth factor" or VEGF) adj5 inhibit*).ti,ab,kf.
29	("anti-vascular" or "antivascular" or "anti-VEGF" or "antiVEGF" or "anti-angiogenic" or "antiangiogenic" or "angiogenesis inhibitor*").ti,ab,kf.
30	exp Angiogenesis Inhibitors/
31	exp Receptors, Vascular Endothelial Growth Factor/
32	(aflibercept or bevacizumab or avastin or ranibizumab or brolucizumab or conbercept or pazopanib or sunitinib or sorafenib or regorafenib or cabozatinib or lenvatinib or ponatinib or axitinib or tivozanib or ramucirumab or vandetanib or sitravatinib).mp.
33	or/27-32 [VEGFi ; angiogenesis inhibitors]
34	26 and 33
35	exp clinical trial/
36	CLINICAL TRIALS AS TOPIC/
37	trial.ti. or (clinical* adj10 trial*).ab. or (phase adj3 study).ti.
38	placebo*.ti,ab.
39	groups.ab.
40	drug therapy.fs.
41	RESEARCH DESIGN/

42	CONTROL GROUPS/
43	Double-Blind Method/
44	single-blind method/
45	Random Allocation/
46	(randomized or randomised or randomly).ti,ab.
47	Prospective Studies/
48	prospective*.ti,ab.
49	or/35-48 [Clinical trials, prospective studies]
50	34 and 49
51	case reports.pt. not (exp clinical study/ or comparative study/ or evaluation studies/ or meta-analysis/ or multicenter study/ or validation studies/ or letter.pt.)
52	case report*.ti,jn.
53	Retrospective Studies/ or retrospective study.ti.
54	51 or 52 or 53
55	50 not 54 [Remove retrospective study and some case reports]
56	limit 55 to "review articles"
57	55 not 56 [remove reviews]
