# Supplementary material for: Adverse events of immune checkpoint therapy alone versus when combined with vascular endothelial growth factor inhibitors: a pooled meta-analysis of 1735 patients
Source: Front Oncol. 2024 Jan 4;13:1238517. doi: 10.3389/fonc.2023.1238517 (PMC10796151; doi:10.3389/fonc.2023.1238517)
Supplement: Supplementary file 2 [file Table_2.pdf]

Supplementary Table S2. Ovid Embase search strategy

#	Searches
1	exp malignant neoplasm/
2	(cancer* or carcinom* or tumor* or tumour* or neoplas* or malignan* or metasta* or myeloma* or leukemia* or lymphoma* or sarcoma* or melanoma* or " myelodysplastic syndrome*" or "stem cell transplant*").ti,ab,kf.
3	1 or 2 [Cancer]
4	("immunotherapy" or "immune therapy" or "immunologic therapy" or "immune checkpoint therapy").ti,ab.
5	(immun* adj3 checkpoint adj3 (inhibitor* or modulator* or antibod* or block*)).ti,ab,kf.
6	(("cytotoxic T lymphocyte associated" adj3 "4") or "CTLA 4" or CTLA4).ti,kf.
7	("Cytotoxic t-lymphocyte antigen" adj3 "4").ti,ab,kf.
8	(ipilimumab or Yervoy).mp.
9	(tremelimumab or ticilimumab).mp.
10	("Programmed Cell Death 1" or PD1 or "PD 1").ti,kf,kw,hw,du,tn.
11	(pembrolizumab or keytruda or lambrolizumab).mp.
12	(nivolumab or opdivo).mp.
13	(spartalizumab* or cetrelimab* or JNJ-63723283).mp.
14	("programmed death ligand 1" or "PD L1" or PDL1 or "PDL-1").ti,kf,kw,hw.
15	(atezolizumab or Tecentriq or durvalumab or imfinzi or avelumab or Bavencio or cemiplimab or libtayo or REGN2810 or "REGN 2810").mp.
16	monalizumab.mp.
17	or/4-16 [immunotherapy checkpoint inhibitors]
18	3 and 17
19	limit 18 to english language
20	Human/
21	Nonhuman/ or ANIMAL/ or Animal Experiment/
22	21 not 20
23	19 not 22
24	(mice or mouse or murine or rat or rats or rodent or cells or "in vitro" or "cell line").ti.
25	23 not 24 [Remove animal or in vitro studies]
26	exp vasculotropin/
27	(("vascular endothelial growth factor" or VEGF) adj5 inhibit*).ti,ab,kf.
28	("anti-vascular" or "antivascular" or "anti-VEGF" or "antiVEGF" or "anti-angiogenic" or "antiangiogenic" or "angiogenesis inhibitor*").ti,ab,kf.
29	exp angiogenesis inhibitor/
30	exp vasculotropin receptor/
31	(aflibercept or bevacizumab or avastin or ranibizumab or brolucizumab or conbercept or pazopanib or sunitinib or sorafenib or regorafenib or cabozatinib or lenvatinib or ponatinib or axitinib or tivozanib or ramucirumab or vandetanib or sitravatinib).mp.
32	or/26-31 [VEGFi ; angiogenesis inhibitors]
33	25 and 32
34	exp clinical trial/
35	double blind procedure/
36	single blind procedure/
37	randomization/
38	(randomized or randomised or randomly).ti,ab.
39	Controlled Study/
40	placebo.ti,ab.
41	groups.ab.
42	trial.ti. or (clinical* adj10 trial*).ab. or (phase adj3 study).ti.
43	Prospective Studies/

44	prospective*.ti,ab.
45	or/34-44 [Clinical trials, prospective studies]
46	33 and 45
47	Case report/
48	Case report*.ti,jx.
49	retrospective study/ or retrospective study.ti.
50	47 or 48 or 49
51	46 not 50 [Remove retrospective study and some case reports]
52	"review"/
53	51 not 52
