# Supplementary material for: Adverse events of immune checkpoint therapy alone versus when combined with vascular endothelial growth factor inhibitors: a pooled meta-analysis of 1735 patients
Source: Front Oncol. 2024 Jan 4;13:1238517. doi: 10.3389/fonc.2023.1238517 (PMC10796151; doi:10.3389/fonc.2023.1238517)
Supplement: Supplementary file 3 [file Table_3.pdf]

Supplementary Table S3. Clarivate Web of Science search strategy

#	Searches
#22	#20 NOT #21
#21	#18 NOT #19 and Review Article (Document Types)
#20	#18 NOT #19
#19	TI= ("case report*" or "retrospective study")
#18	#17 AND #16
#17	TS= clinical trial* OR TS=research design OR TS=comparative stud* OR TS=evaluation stud* OR TS=controlled trial* OR TS=follow-up stud* OR TS=prospective stud* OR TS=random* OR TS=placebo* OR TS=(single blind*) OR TS=(double blind*) OR TS="control group*" OR TI= trial OR TI=(phase NEAR3 Study)
#16	#15 AND #11
#15	#12 OR #13 OR #14
#14	TS=((aflibercept or bevacizumab or avastin or ranibizumab or brolucizumab or conbercept or pazopanib or sunitinib or sorafenib or regorafenib or cabozatinib or lenvatinib or ponatinib or axitinib or tivozanib or ramucirumab or vandetanib or sitravatinib))
#13	TS=(("anti-vascular" or "antivascular" or "anti-VEGF*" or "antiVEGF*" or "anti-angiogenic" or "antiangiogenic" or "angiogenesis inhibitor*"))
#12	TS=((("vascular endothelial growth factor" or VEGF* or angiogenesis) NEAR/5 inhibit*))
#11	#9 NOT #10
#10	TI=(mice or mouse or murine or rat or rats or rodent or cells or "in vitro" or "cell line")
#9	#1 AND #8
#8	#2 OR #3 OR #4 OR #5 OR #6 OR #7
#7	TS=((ipilimumab or Yervoy or tremelimumab or ticilimumab or pembrolizumab or keytruda or lambrolizumab or nivolumab or opdivo or spartalizumab or cetrelimab or "JNJ-63723283" or atezolizumab or Tecentriq or durvalumab or imfinzi or avelumab or Bavencio or cemiplimab or libtayo or REGN2810 or "REGN 2810" or monalizumab))
#6	TS=("CTLA 4" or CTLA4 or "Programmed Cell Death 1" or PD1 or "PD 1" or "programmed death ligand 1" or "PD L1" or PDL1 or PDL-1)
#5	TI=("cytotoxic T lymphocyte associated" or "cytotoxic T-lymphocyte antigen*")
#4	TS=((("cytotoxic T lymphocyte associated" or "cytotoxic T-lymphocyte antigen") NEAR/3 "4"))
#3	TS=((checkpoint NEAR/3 (inhibitor* or modulator* or antibod* or block*)))
#2	TS=(("immunotherapy" or "immune therapy" or "immunologic therapy" or "immune checkpoint therapy"))
#1	TS=((cancer* or carcinom* or tumor* or tumour* or neoplas* or malignan* or metasta* or myeloma* or leukemia* or leukaemia* or lymphoma* or sarcoma* or melanoma* or "myelodysplastic syndrome*" or "stem cell transplant*"))
