# Supplementary material for: Adverse events of immune checkpoint therapy alone versus when combined with vascular endothelial growth factor inhibitors: a pooled meta-analysis of 1735 patients
Source: Front Oncol. 2024 Jan 4;13:1238517. doi: 10.3389/fonc.2023.1238517 (PMC10796151; doi:10.3389/fonc.2023.1238517)
Supplement: Supplementary file 4 [file Table_4.pdf]

Supplementary Table S4. Wiley Cochrane Library search strategy

ID	Search
#1	MeSH descriptor: [Neoplasms] explode all trees
#2	((cancer* or carcinom* or tumor* or tumour* or neoplas* or malignan* or metasta* or myeloma* or leuk?emia* or lymphoma* or sarcoma* or melanoma* or " myelodysplastic syndrome*" or "stem cell transplant*")):ti,ab,kw (Word variations have been searched)
#3	#1 OR #2
#4	(("immunotherapy" or "immune therapy" or "immunologic therapy" or "immune checkpoint therapy")):ti,ab,kw (Word variations have been searched)
#5	((checkpoint NEAR/3 (inhibitor* or modulator* or antibod* or block*))):ti,ab,kw (Word variations have been searched)
#6	((("cytotoxic T lymphocyte associated" NEAR/3 "4") or "CTLA 4" or CTLA4)):ti,ab,kw (Word variations have been searched)
#7	(("Cytotoxic t-lymphocyte antigen" NEAR/3 "4")):ti,ab,kw (Word variations have been searched)
#8	(("cytotoxic T lymphocyte associated" or "cytotoxic T-lymphocyte antigen*")):ti (Word variations have been searched)
#9	(("CTLA 4" or CTLA4 or "Programmed Cell Death 1" or PD1 or "PD 1" or "programmed death ligand 1" or "PD L1" or PDL1 or PDL-1)):ti,ab,kw (Word variations have been searched)
#10	((ipilimumab or Yervoy or tremelimumab or ticilimumab or pembrolizumab or keytruda or lambrolizumab or nivolumab or opdivo or spartalizumab or cetrelimab or "JNJ-63723283" or atezolizumab or Tecentriq or durvalumab or imfinzi or avelumab or Bavencio or cemiplimab or libtayo or REGN2810 or "REGN 2810" or monalizumab)):ti,ab,kw (Word variations have been searched)
#11	{OR #4-#10}
#12	#3 AND #11
#13	((mice or mouse or murine or rat or rats or rodent or cells or "in vitro" or "cell line")):ti (Word variations have been searched)
#14	#12 NOT #13
#15	((("vascular endothelial growth factor" or VEGF* or angiogenesis) NEAR/5 inhibit*)):ti,ab,kw (Word variations have been searched)
#16	(("anti-vascular" or "antivascular" or "anti-VEGF*" or "antiVEGF*" or "anti-angiogenic" or "antiangiogenic" or "angiogenesis inhibitor*")):ti,ab,kw (Word variations have been searched)
#17	((aflibercept or bevacizumab or avastin or ranibizumab or brolucizumab or conbercept or pazopanib or sunitinib or sorafenib or regorafenib or cabozatinib or lenvatinib or ponatinib or axitinib or tivozanib or ramucirumab or vandetanib or sitravatinib)):ti,ab,kw (Word variations have been searched)
#18	{OR #15-#17}
#19	#14 AND #18
