# Supplementary material for: Adverse events of immune checkpoint therapy alone versus when combined with vascular endothelial growth factor inhibitors: a pooled meta-analysis of 1735 patients
Source: Front Oncol. 2024 Jan 4;13:1238517. doi: 10.3389/fonc.2023.1238517 (PMC10796151; doi:10.3389/fonc.2023.1238517)
Supplement: Supplementary file 6 [file Table_6.pdf]

Supplementary Table S6. Baseline studies characteristics

Author	Trial phase	Randomization	Blinding		Drug(s)	Number of patients	Median age	Males, %
Bendell et al.	3	Yes	No	ICT	atezolizumab	90	56	59%
				ICT+VEGFi	atezolizumab + cobimetinib	183	58	58%
McDermott et al.	2	Yes	No	ICT	atezolizumab	103	61	77
				ICT+VEGFi	atezolizumab + bevacizumab	101	62	74
Lonardi et al.	2	Yes	No	ICT	avelumab	30	65	20
				ICT+VEGFi	avelumab + cetuximab	30	63	43
Loriot et al.	3	Yes	Yes	ICT	pembrolizumab	223	73	
				ICT+VEGFi	pembrolizumab + lenvatinib	218	74	
Nayak	2	Yes	No	ICT	pembrolizumab	30	55	
				ICT+VEGFi	pembrolizumab + bevacizumab	50	52	
Yang et al.	3	Yes	Yes	ICT	pembrolizumab	314		
				ICT+VEGFi	pembrolizumab + lenvatinib	309		
Lheureux et al.	2	Yes	No	ICT	nivolumab	18	67	
				ICT+VEGFi	nivolumab + carbozatinib	36	66	

ICT – immune checkpoint inhibitors therapy; VEGFi - vascular endothelial growth factor inhibitors
